# Applicability of Traps for Collecting Mosquito Immatures (Diptera: Culicidae) for Entomological Surveillance of Arbovirus Vectors in a Remnant of the Atlantic Forest, Rio de Janeiro State, Brazil

**DOI:** 10.3390/tropicalmed9060125

**Published:** 2024-05-29

**Authors:** Rayane Dias, Cecilia Ferreira de Mello, Shayenne Olsson Freitas Silva, Hélcio Reinaldo Gil-Santana, Ana Laura Carbajal-de-la-Fuente, Jeronimo Alencar

**Affiliations:** 1 Laboratorio de Diptera, Instituto Oswaldo Cruz (Fiocruz), Avenida Brasil 4365, Manguinhos, Rio de Janeiro 21040-360, Brazil; rayanedias@aluno.fiocruz.br (R.D.); cecilia.mello@ioc.focruz.br (C.F.d.M.); shayennesilva@ioc.fiocruz.br (S.O.F.S.); helciogil@ioc.fiocruz.br (H.R.G.-S.); 2 Programa de Pós-Graduação em Medicina Tropical, Instituto Oswaldo Cruz (Fiocruz), Avenida Brasil 4365, Manguinhos, Rio de Janeiro 21040-360, Brazil; 3 Consejo Nacional de Investigaciones Científicas y Técnicas (CONICET), Buenos Aires C1063, Argentina; carbajal@conicet.gov.ar; 4 Centro Nacional de Diagnóstico e Investigación en Endemo-Epidemias (CeNDIE), Administración Nacional de Laboratorios e Institutos de Salud “Dr. Carlos Malbrán” (ANLIS), Av. Paseo Colón 568, Buenos Aires C1063, Argentina

**Keywords:** Culicidae, immature forms, *Haemagogus*, larval habitat, vector surveillance

## Abstract

Diverse larval habitats significantly influence female mosquito oviposition. Utilizing traps that simulate these habitats is helpful in the study of the bioecology and characteristics of pathogen-transmitting species during oviposition. This study evaluated the feasibility of different traps in natural environments by comparing sampling methods and detecting the oviposition of epidemiologically important mosquitoes, with emphasis on *Haemagogus* species, in a fragment of the Atlantic Forest in Silva Jardim, Rio de Janeiro State, Brazil. Monthly collections were conducted from March 2021 to October 2023 using four types of traps: plastic containers, tires, bamboo, and sapucaia. Immatures were collected from these traps using a pipette, placed in plastic bags, and transported to the laboratory. Tire was the most efficient trap, showing the highest mosquito abundance (n = 1239) and number of species (S = 11). Conversely, the plastic container trap exhibited the lowest diversity (H = 0.43), with only two species and a low mosquito abundance (n = 26). The bamboo trap captured six species and recorded the second-highest diversity index (H = 1.04), while the sapucaia trap captured five species and had the third-highest diversity index (H = 0.91). Of the total immatures collected, 1817 reached adulthood, comprising 13 species, two of which are vectors of the sylvatic yellow fever virus: *Haemagogus leucocelaenus* and *Haemagogus janthinomys*. In conclusion, detecting key vectors of the sylvatic yellow fever virus in Brazil highlights the need for ongoing entomological and epidemiological surveillance in the study area and its vicinity. These efforts are crucial for monitoring vector presence and activity, identifying potential transmission hotspots, and devising effective control and prevention strategies.

## 1. Introduction

Culicidae, insects widely distributed in urban and forest environments, depend on water collections to ensure the successful development of their immature stages (egg, larva, and pupa). These aquatic environments serve as habitats for the immature forms, which, in addition to providing water for oviposition and the continuation of immature phases, are also influenced by chemical and physical factors [1].

Immature habitats are categorized as either natural or artificial. Natural habitats typically include ponds, wetlands, tree hollows, bromeliads, and bamboo, whereas artificial habitats comprise containers that accumulate water, such as dams, water tanks, tires, and cans [2]. Female mosquitoes are selective about their breeding sites. Specifically, those of the genus *Haemagogus* prefer temporary sites in forest environments, often colonizing tree and bamboo hollows, with records also in bamboo and bromeliad internodes [3,4,5].

Using traps for oviposition by female mosquitoes facilitates data collection on species richness, dominance, abundance, and diversity. These traps also serve as tools for taxonomic studies critical for epidemiological surveillance in regions affected by arboviruses, such as the sylvatic yellow fever virus (YFV). Among the species identified as transmitters of YFV, *Haemagogus janthinomys* Dyar, 1921 is recognized as the primary vector in the Americas, while *Haemagogus leucocelaenus* Dyar and Shannon, 1924 was implicated in the recent YFV outbreak from 2016 to 2018 in Rio de Janeiro State, Brazil [6]. During epidemic periods, these species often have high infection rates in tree-canopy samples [7].

The present study aimed to evaluate the feasibility of various types of traps introduced into the natural environment, compare their efficiency, and detect the oviposition of mosquito species of epidemiological importance, with a focus on *Haemagogus* species in a segment of the Atlantic Forest located in the municipality of Silva Jardim, Rio de Janeiro State.

## 2. Material and Methods

### 2.1. Ethics Statement

The permanent license for the collection, capture, and transport of zoological material was granted by the Chico Mendes Institute for Biodiversity Conservation (ICMBio) and the Biodiversity Authorization and Information System (SISBIO) under license number 44333-1, Rio de Janeiro, Brazil. All team members were properly vaccinated against yellow fever.

### 2.2. Study Area

**Figure 1:**
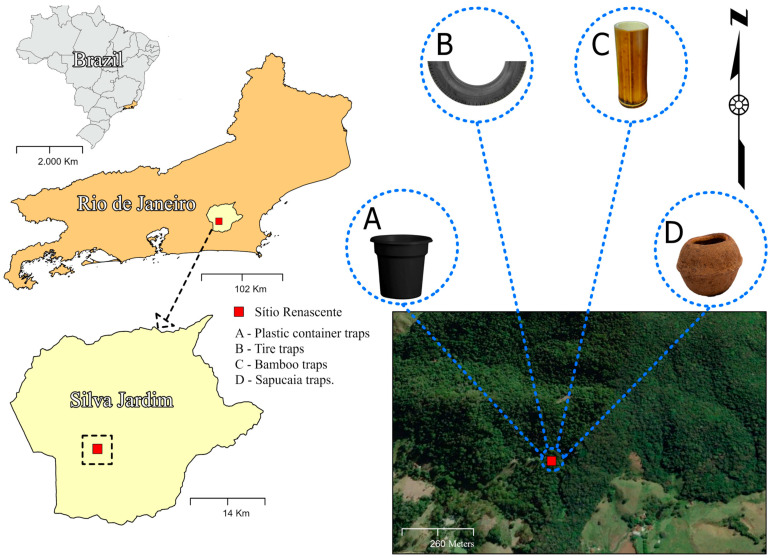
Map of the study site in the municipality of Silva Jardim, Rio de Janeiro State, Brazil.

The sampling area is situated within a remnant of the Atlantic Forest, located on the boundaries of the Renascente site in the central–north region of Rio de Janeiro, specifically in Silva Jardim. This region still preserves more than 18.70% of the original Atlantic Forest coverage (Fundação SOS Mata Atlântica, 2019). Sub-mountain dense ombrophilous forest is the predominant vegetation [8]. The regional climate is categorized as a rainy tropical climate with a dry season in winter, according to the Köppen classification. Annual rainfall ranges from 1500 to 2000 mm, with the period from November to March experiencing the highest rainfall and temperatures, and varies annually between 32 and 24 °C [9]. The collection site was located at the geographical coordinates 22°36′49.1″ S 42°27′34.1″ W (Figure 1).

### 2.3. Experimental Design

Collections were conducted monthly from March 2021 to October 2023. The traps for collecting mosquito immatures were characterized as follows. (a) The plastic container traps consisted of a matte black container with a 500 mL water capacity. The container had no lid and was filled with natural water and forest litter (leaves, branches, and other organic materials) to mimic a natural ecosystem. (b) The tire traps were made from a third of a motorcycle tire; these traps served as containers with 1000 mL of water capacity. (c) The bamboo traps were constructed from a bamboo internode; these traps were separated to create a container about 30 cm deep, with an opening approximately 25 cm in diameter and a 1000 mL water capacity. (d) The sapucaia traps were made from the woody fruit of the Atlantic Forest chestnut tree (“monkey-cumbuca”); these traps were 12 to 20 cm in diameter with an operculum or dehiscent lid. For the experiment, a fruit capable of holding 500 mL of water was selected, occupying two-thirds of the fruit’s volume and matching the water volume in the other traps. To collect immature mosquitoes, 500 mL of water were added to each trap. The traps were installed upright and tied to the trunks of four trees at the same location and positioned 2 m from the ground level.

The larvae and pupae found in the traps were collected using a plastic pipette, transferred to 250 mL plastic bags (Whirl-Pak^®^ bags, BioQuip^®^, Seattle, WA, USA), and transported to the laboratory. In the laboratory, they were maintained alive in pots containing water from the reservoir in which they were collected, supplemented with dechlorinated water as needed due to evaporation until they reached the adult phase. They were kept in an air-conditioned B.O.D. chamber (ELETROlab, EL131/3, capacity: 354 L, São Paulo, Brazil) in controlled conditions, with a temperature of 28 ± 1 °C, relative humidity from 75% to 90%, and a photoperiod of 12 h. The larvae were fed twice weekly with fish food (TetraMin^®^, Melle, Germany), which was ground and diluted in 10 mL of water.

In the laboratory, the tire, bamboo, plastic container, and sapucaia traps were immersed in plastic basins filled with distilled water to facilitate any potential hatching of the eggs deposited on their walls. Immatures reaching the adult stage were euthanized by intoxication using a solution of ethyl ether or chloroform.

Culicids were identified by direct observation of the morphological characters evident under a stereoscopic microscope (Leica DMD108^®^, Wetzlar, Germany), based on the dichotomous keys by Lane (1953) [10,11], Faran and Linthicum (1981) [12], Consoli and Lourenço-de-Oliveira (1994) [13], and Forattini (2002) [3]. Following identification, they were deposited in the Entomological Collection of the Oswaldo Cruz Institute, FIOCRUZ, under the title “Mata Atlântica/RJ”.

### 2.4. Statistical Analyses

Several statistical analyses were conducted to assess the structure of the mosquito community. These included the Shannon diversity index (H), which serves as an indicator of both species richness and evenness; Pielou’s equitability (J), which assesses the equitable distribution of species abundances; species richness (S), to represent the total number of distinct species; absolute abundance of individuals, which indicates the overall count of the mosquitoes; and the dominance index (D), which reveals prevalent patterns within the community. Additionally, the Morisita similarity index was used to quantify the level of resemblance between different mosquito breeding sites. This index aids in elucidating the patterns of species distribution and composition across diverse habitats. All statistical analyses were performed using the software PAST version 4.05, a robust analytical platform for exploring ecological data [14].

## 3. Results

**Figure 2:**
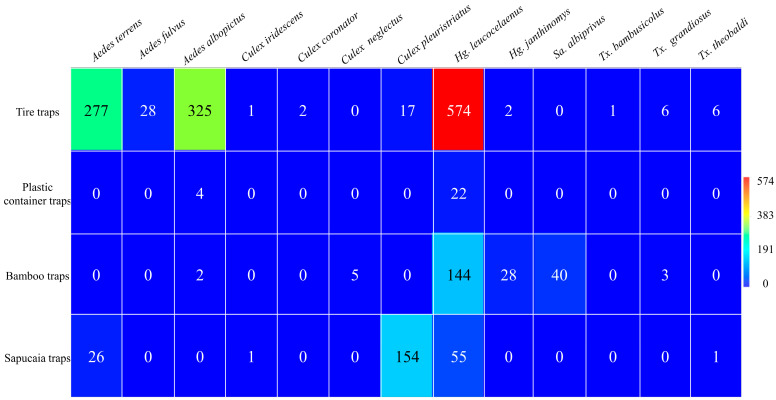
Abundance of mosquitoes collected from different traps at Sítio Renascente, Silva Jardim, Rio de Janeiro State, Brazil, from March 2021 to October 2023.

During the sampling period, 2016 immatures were collected, of which 1723 reached adulthood in the types of traps used. The greatest abundance was found in tire traps (n = 1239; 72%), followed by sapucaia traps (n = 237; 14%), bamboo traps (n = 222; 13%), and plastic container traps (n = 26; 2%). The mosquito species recorded and their respective abundances included *Aedes terrens* Walker, 1856 (n = 303; 17.6%); *Aedes fulvus* Wiedemann, 1828 (n = 28; 2%); *Aedes albopictus* Skuse, 1894 (n = 331; 19.2%); *Culex iridescens* Lutz, 1905 (n = 2; 0.1%); *Culex coronator* Dyar and Knab, 1906 (n = 2; 0.1%); *Culex neglectus* Lutz, 1904 (n = 5; 0.3%); *Culex pleuristriatus* Theobald, 1903 (n = 171; 9.9%); *Haemagogus leucocelaenus* (n = 795; 46.1%); *Haemagogus janthinomys* (n = 30; 1.7%); *Sabethes albiprivus* Theobald, 1903 (n = 40; 2.3%); *Toxorhynchites bambusicolus* Lutz and Neiva 1913 (n = 1; 0.1%); *Toxorhynchites* cf. *grandiosus* Williston 1900 (n = 9; 0.5%); and *Toxorhynchites* cf. *theobaldi* Dyar and Knab, 1906 (n = 70; 4%) (Figure 2).

**Figure 3:**
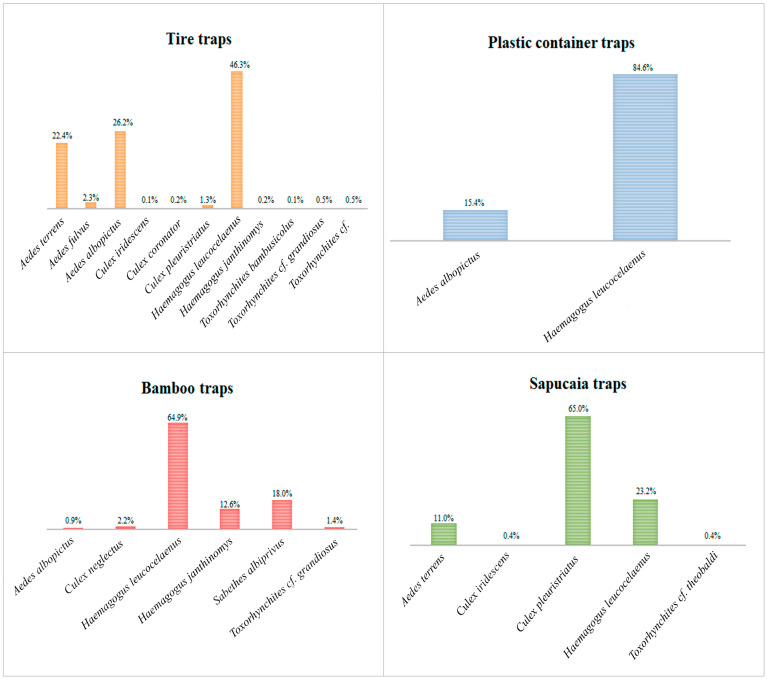
Percentage of the abundance of each species per trap, considering all Culicidae species collected at Sítio Renascente, Silva Jardim, Rio de Janeiro State, Brazil, from March 2021 to October 2023.

Analyzing the species separately by trap, we observed that *Hg. leucocelaenus* exhibited greater abundance in all traps, except the sapucaia traps, where it was the second most abundant species. *Haemagogus leucocelaenus* accounted for 85% of the total adult specimens in the plastic container traps, 65% in the bamboo traps, 46% in the tire traps, and 23% in the sapucaia traps. Meanwhile, *Hg. janthinomys* was present in the bamboo and tire traps, representing 13% and 0.2% of their respective populations (Figure 3).

Tires stood out as the most prolific traps, exhibiting the highest abundance of mosquitoes and species richness (S = 11). Additionally, they presented a high diversity index (H = 1.27), indicating a wide variety of species present. However, their equitability was relatively low (J = 0.53), suggesting an unequal distribution among species. In contrast, the plastic container traps had the lowest diversity (H = 0.43), registering only two species and a low mosquito abundance (26 individuals). These traps showed a considerable dominance index (D = 0.73), with most specimens belonging to *Hg. leucocelaenus* (85%). Six species were reported in the bamboo traps, presenting the second-highest diversity index (H = 1.04). Finally, five species were recorded in the sapucaia traps, which exhibited the third-highest diversity index (H = 0.91) (Table 1, Supplementary File S1).

**Figure 4:**
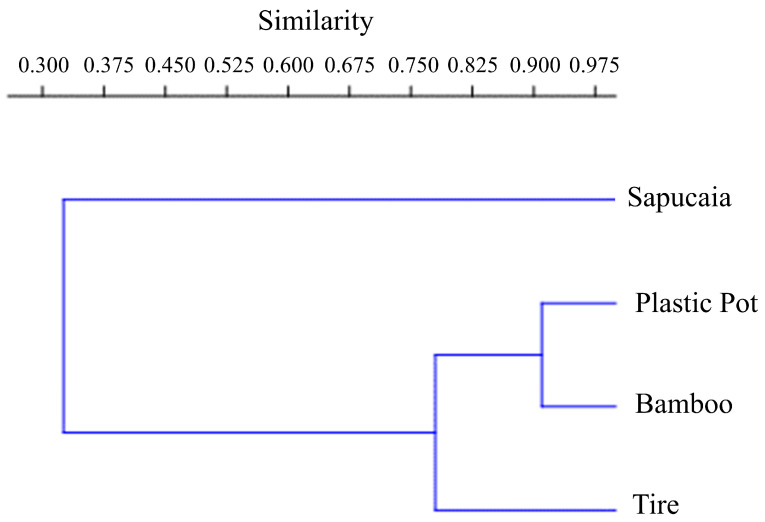
Morisita index cladogram of similarity between mosquito immature traps at Sítio Renascente, Silva Jardim, Rio de Janeiro State, Brazil, from March 2021 to October 2023.

According to the Morisita similarity index, the sapucaia traps were the most divergent, differing significantly from the others. These recorded a higher number of *Cx. pleuristriatus* individuals. The most similar traps were the plastic container and the bamboo traps, both featuring similar equitability indices (bamboo traps, J = 0.5786; plastic container traps J = 0.6194) (Figure 4). Both types of traps predominantly captured *Hg. leucocelaenus*, accounting for 85% of the Culicidae in the plastic container traps and 65% in the bamboo traps.

## 4. Discussion

Culicidae immatures inhabit a variety of water-collecting environments, featuring diverse substrates with varying degrees of human influence. This plasticity highlights the importance of conducting studies aimed at evaluating, characterizing, and defining different ecological variables across various traps. Several species exhibit significant ecological flexibility, enabling them to thrive in varied environments that support their progression from immature to adult stages [3].

Maia et al. (2020) [15] employed three types of traps akin to those in this study: tire, bamboo, and plastic container traps. The tire traps captured the largest number of individuals, which is in line with our observations. However, there was a variance in the most abundant species; their study reported significantly lower proportions of *Hg. leucocelaenus* (5.90% of total individuals at collection point 1 and 1.30% at collection point 2), compared to our findings, which indicated a much higher prevalence, nearly 47%.

We found that tires were the most effective traps, capturing the highest abundance of immature Culicidae and the largest number of *Hg. leucocelaenus*. In Brazil, similar studies comparing natural and artificial traps in the rural areas of northern Paraná also reported the highest mosquito abundance in tire traps [16,17]. *Aedes albopictus* was the second most abundant species in the tire traps, a similar finding to that of Albuquerque et al. (2000) [18], who noted a high abundance of *Ae. albopictus* in tires in a remnant of the Atlantic Forest in the urban area of Recife, Pernambuco. In our study, the species found with the highest abundance in the tire traps were *Hg. leucocelaenus* (46%), *Ae. albopictus* (26%), and *Ae. terrens* (22%). These results contrast with those from Nova Iguaçu, Rio de Janeiro, where the most abundant species in the tire traps were *Limatus durhamii* Theobald, 1901 (57.8%) and *Limatus pseudomethisticus* Bonne-Wepster and Bonner, 1920 (25.7%), and in Londrina, Paraná, where the predominant species in the tire traps were *Culex eduardoi* Casal and García, 1968, *Culex laticlasper* Galindo and Blanton, 1954, *Culex bigoti* Bellardi, 1862, and *Culex quinquefasciatus* Say, 1823 [15,16].

In the present study, the bamboo traps exhibited the second-highest index of Culicidae diversity. These results are in line with those from a study conducted on live bamboo internodes in Nagasaki, Japan, where the most dominant arthropod groups were immature stages of Diptera from the families Ceratopogonidae and Culicidae [19]. The prevalence of immature Culicidae in bamboo underscores the role of plants as habitats for various mosquito species, including those of public health relevance. Our study revealed a significant abundance of *Hg. leucocelaenus* (65%) in bamboo traps, underscoring its relevance as a medically important species and a YFV vector [20,21].

The high diversity observed in bamboo traps is also supported by Bastos et al. (2021) [22]. They conducted a study on the composition of Culicidae that breed in internodes of *Bambusa* sp., finding a richness of 17 species in a remnant of Atlantic Forest in Rio de Janeiro State. In our study, six species were recorded in this trap type, the most abundant being *Hg. leucocelaenus* (65%), *Sa. albiprivus* (18%), and *Hg. janthinomys* (13%). These genera are of extreme medical significance, as they are the principal sylvatic vectors of YFV in the Americas [4,7,23].

We observed that the plastic container and bamboo traps showed high similarity, as evidenced by the Morisita index. This similarity was further supported by comparable equitability indices, indicating an equitable distribution of the species present. Additionally, both traps exhibited a high abundance of *Hg. leucocelaenus* and *Ae. albopictus*, suggesting that they may serve as favorable breeding sites for these pathogen-transmitting vectors. These traps provide similar and favorable environmental conditions for Culicidae proliferation. They feature a cylindrical shape, which offers good water storage capacity, potentially reducing evaporation. Moreover, both can accumulate organic debris, such as leaves and plant remains, serving as a food source for immature forms.

The presence of other traps installed at the sampling point may have influenced oviposition, as these species already recognize them as potentially advantageous larval breeding habitats. The two species found most abundantly in these traps (plastic container and bamboo traps) display strong adaptive capacity, enabling them to colonize traps in both forest environments and those modified by human activity [4].

Marques and Forattini (2008) [24] noted that the abundance of species reflects the control one species exerts over others to become dominant. The dominance of *Hg. leucocelaenus* and *Ae. albopictus*, found in nearly all traps, is likely due to their biological, ecological, and behavioral similarities. Both species are sylvatic, laying their eggs on moist substrates near the water surface; they are typically found in tropical and subtropical forest environments, with *Ae. albopictus* also prevalent in rural areas [3,25,26,27].

Understanding the diversity of insects in the Atlantic Forest is crucial for assessing changes in population activity patterns. Our results suggest that sampling methods for collecting immature mosquitoes are significant indicators in this regard. Tire traps showed the highest abundance and richness of Culicidae, underscoring their importance in projects monitoring biological vectors relevant to public health. The increase in production and improper disposal of materials such as tires significantly contributes to the creation of mosquito breeding sites. *Aedes aegypti* Linnaeus, 1762, a vector for diseases like dengue, Zika, and chikungunya, notably thrives in these environments. Tires are particularly effective breeding sites for mosquitoes due to their water retention capacity and protection for larval development. Moreover, they can act as passive carriers of mosquito eggs and larvae, potentially being moved by rainwater or human actions, thus spreading mosquitoes to new areas and contributing to disease transmission [28]. Consequently, tire traps for collecting immature mosquitoes are valuable tools in fauna surveys, functioning as artificial phytotelmata [29].

## 5. Conclusions

The observed oviposition behavior of mosquito species of the genus *Haemagogus*, specifically *Hg. leucocelaenus* and *Hg. Janthinomys,* demonstrates their capacity to lay eggs in artificial containers. This behavior increases the risk of contact between humans and vectors at the study site. Consequently, using various traps for sampling is an effective approach for surveying Culicidae fauna, enabling studies focused on the surveillance and monitoring of medically important vectors.

## Figures and Tables

**Table 1:** Ecological indices of the different traps installed at Sítio Renascente, Silva Jardim, Rio de Janeiro State, Brazil, from March 2021 to October 2023.

Ecological Index	Trap Type			
	Tire	Plastic Container	Bamboo	Sapucaia
Abundance	1239	26	222	237
Species (S)	11	2	6	5
Dominance (D)	0.3342	0.7396	0.4699	0.49
Shannon Diversity (H)	1.27	0.4293	1.04	0.91
Equitability (J)	0.53	0.6194	0.5786	0.564

## Data Availability

Not applicable.

## Supplementary Materials

The following supporting information can be downloaded at: https://www.mdpi.com/article/10.3390/tropicalmed9060125/s1. Supplementary File S1: Diversity and abundance of species mosquitoes collected from different traps at Sítio Renascente, Silva Jardim, Rio de Janeiro State, Brazil, from March 2021 to October 2023.
