# Supplementary material for: Applicability of Traps for Collecting Mosquito Immatures (Diptera: Culicidae) for Entomological Surveillance of Arbovirus Vectors in a Remnant of the Atlantic Forest, Rio de Janeiro State, Brazil
Source: Trop Med Infect Dis. 2024 May 29;9(6):125. doi: 10.3390/tropicalmed9060125 (PMC11209091; doi:10.3390/tropicalmed9060125)
Supplement: Supplementary file 1 [file tropicalmed-09-00125-s001.zip › tropicalmed-2987709-Supplementary File S1.pdf]

Compare breeders in terms of Diversity and Abundance of species at Sítio Renescente

Species	Tire	Plastic countainer	Bamboo	Sapucaia	TOTAL	
Aedes terrens	277	0	0	26	303	18%
Aedes fulvus	28	0	0	0	28	2%
Aedes albopictus	325	4	2	0	331	19%
Culex iridescens	1	0	0	1	2	0%
Culex coronator	2	0	0	0	2	0%
Culex neglectus	0	0	5	0	5	0%
Culex pleuristriatus	17	0	0	154	171	10%
Haemogogus leucocelaenus	574	22	144	55	795	46%
Haemagogus janthynomys	2	0	28	0	30	2%
Sabethes albiprivus	0	0	40	0	40	2%
Toxorhynchites bambusicolus	1	0	0	0	1	0%
Toxorhynchites cf. grandiosus	6	0	3	0	9	1%
Toxorhynchites cf. theobaldi	6	0	0	1	7	0%
Total	1239	26	222	237	1723	

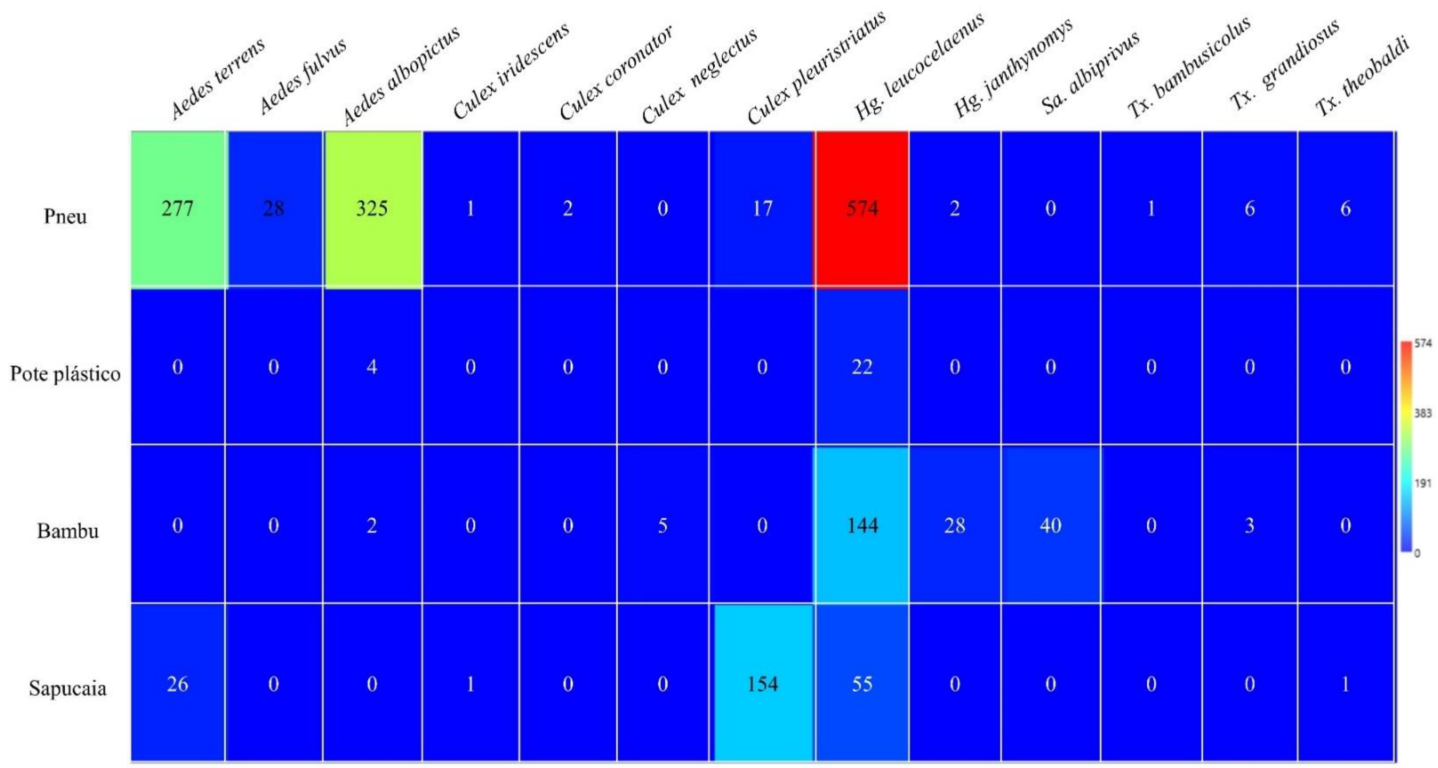

	Tire	Plastic countainer	Bamboo	Sapucaia
**Taxa_S**	11	2	6	5
**Individuals**	1239	26	222	237
**Dominance_D**	0,3342	0,7396	0,4699	0,49
Simpson_1-D	0,6658	0,2604	0,5301	0,51
**Shannon_H**	1,27	0,4293	1,04	0,91
Evenness_e^H/S	0,324	0,7681	0,47	0,4957
Brillouin	1.254	0,3697	0,9931	0,8771
Menhinick	0,3125	0,3922	0,4027	0,3248
Margalef	1.404	0,3069	0,9255	0,7315
**Equitability_J**	0,53	0,6194	0,5786	0,564
Fisher_alpha	1.663	0,505	1.136	0,8957
Berger-Parker	0,4633	0,8462	0,6486	0,6498
Chao-1	11,33	2	6	6

Species	FREQUENCY (PRESENCE OR ABSENCE)					
	Tire	Plastic container	Bamboo	Sapucaia	Yes	No
Aedes terrens	Yes	No	No	Yes	2	2
Aedes fulvus	Yes	No	No	No	1	3
Aedes albopictus	Yes	Yes	Yes	No	3	1
Culex iridescens	Yes	No	No	Yes	2	2
Culex coronator	Yes	No	No	No	1	3
Culex neglectus	No	No	Yes	No	1	3
Culex pleuristriatus	Yes	No	No	Yes	2	2
Haemogogus leucocelaenus	Yes	Yes	Yes	Yes	4	0
Haemagogus janthynomys	Yes	No	Yes	No	2	2
Sabethes albiprivus	No	No	Yes	No	1	3
Toxorhynchites bambusicolus	Yes	No	No	No	1	3
Toxorhynchites cf. grandiosus	Yes	No	Yes	No	2	2
Toxorhynchites cf. theobaldi	Yes	No	No	Yes	2	2
	11	2	6	5		

Total 3 year					
Species	Tire	Plastic countainer	Bamboo	Sapucaia	TOTAL
Aedes terrens (Walker)	277	0	0	26	303
Aedes fulvus Wiedemann, 1828	28	0	0	0	28
Aedes albopictus Skuse	325	4	2	0	331
Culex iridescens Lutz, 1905	1	0	0	1	2
Culex coronator Dyar & Knab, 1906	2	0	0	0	2
Culex neglectus Lutz	0	0	5	0	5
Culex pleuristriatus Theobald	17	0	0	154	171
Culex (Melanoconion.) sp.	0	0	1	71	72
Haemogogus leucocelaenus Dyar & Shannon, 1924	574	22	144	55	795
Haemagogus janthynomys Dyar/capricornii Lutz	2	0	28	0	30
Sabethes albiprivus Theobald, 1903	0	0	40	0	40
Toxorhynchites bambusicolus Lutz & Neiva, 1913	1	0	0	0	1
Toxorhynchites cf. grandiosus Williston, 1900	6	0	3	0	9
Toxorhynchites cf. theobaldi Dyar & Knab, 1906	6	0	0	1	7
Toxorhynchites sp	9	3	5	5	22
	0	0	0	0	0
					1817

Total 3 anos SOMENTES ESPÉCIES					
Species	Tire	Plastic countainer	Bamboo	Sapucaia	TOTAL
Aedes terrens (Walker)	277	0	0	26	303
Aedes fulvus Wiedemann, 1828	28	0	0	0	28
Aedes albopictus Skuse	325	4	2	0	331
Culex iridescens Lutz, 1905	1	0	0	1	2
Culex coronator Dyar & Knab, 1906	2	0	0	0	2
Culex neglectus Lutz	0	0	5	0	5
Culex pleuristriatus Theobald	17	0	0	154	171
Haemogogus leucocelaenus Dyar & Shannon, 1924	574	22	144	55	795
Haemagogus janthynomys Dyar/capricornii Lutz	2	0	28	0	30
Sabethes albiprivus Theobald, 1903	0	0	40	0	40
Toxorhynchites bambusicolus Lutz & Neiva, 1913	1	0	0	0	1
Toxorhynchites cf. grandiosus Williston, 1900	6	0	3	0	9
Toxorhynchites cf. theobaldi Dyar & Knab, 1906	6	0	0	1	7
					1723
